# Alterations in physico-chemical properties, microstructure, sensory characteristics, and volatile compounds of red pepper (*Capsicum annuum* var. conoides) during various thermal drying durations

**DOI:** 10.1016/j.fochx.2024.101566

**Published:** 2024-06-20

**Authors:** Cen Li, Yongjun Wu, Qiyan Zhu, Chuanzheng Xie, Yan Yan

**Affiliations:** a Key Laboratory of Plant Resource Conservation and Germplasm Innovation in Mountainous Region (Ministry of Education), College of Life Sciences/Institute of Agro-bioengineering, Guizhou University, Guiyang 550025, Guizhou Province, China; b Guizhou University, Industrial Technology Institute of Pepper, Guiyang 550025, Guizhou Province, China; c Guizhou Chang Ge Food Co. Ltd., Qiannan 551200, Guizhou Province, China; d School of Liquor and Food Engineering, Guizhou University, Guizhou Province Key Laboratory of Fermentation Engineering and Biopharmacy, Guiyang 550025, Guizhou Province, China

**Keywords:** Pepper, Thermal drying duration, Physico-chemical properties, Sensory characteristics, Volatile compounds profile

## Abstract

Red pepper (*Capsicum annuum* var. conoides) is commonly used for dried pepper production in China, and the drying process, particularly the during duration, profoundly affects its quality. The findings indicate that prolonged exposure to high temperatures during thermal drying results in significant darkening, an evident decrease in red and yellow tones, and gradual transformation of the pepper's microscopic structure from granular to compact, along with 88% reduction in moisture content and 81% decrease in thickness. The capsaicinoid content increased, resulting in a 4.3-fold increase in spiciness after drying compared to that of fresh pepper. The pepper aroma shifts from fruity, choking, and grassy to herb, dry wood, and smoky. Compounds such as 2-Acetylfuran, furfural, 2-methylfuran, 1-methylpyrrole, 2-methylpyrazine, and 2,5-dimethylpyrazine exhibited positive correlations with drying time, whereas ethyl 2-methylpropanoate, ethyl butanoate, ethyl 2-methylbutanoate, ethyl hexanoate, and 3-methylbutyl butanoate showed negative correlations, indicating their potential as markers for monitoring thermal drying processes.

## Highlights

• A systematic study of red pepper during various thermal drying durations.
• The spiciness increases notably as water is lost.
• The aroma shifts from fruity, choking, and grassy to herb, dry wood, and smoky.
• The potential markers for monitoring thermal drying processes of red pepper.

## 1 Introduction

Peppers (*Capsicum annuum*) are known for their nutritional value, vibrant color, and distinctive flavor and are cultivated across all continents. China is the world's largest producer of fresh peppers (Zhang et al., 2023). Notably, *Capsicum annuum* var. conoides is among the most extensively cultivated pepper types by land area. The significance of pepper lies predominantly in its color and flavor; flavor plays a pivotal role as the primary determinant of quality, which subsequently affects the market value. Fresh red peppers with high moisture content are susceptible to spoilage during sale, transportation, and storage; therefore, they are often dried to extend their shelf life (Mun et al., 2024). Dried peppers inhibit microbial growth and reproduction, which extends their shelf life and facilitates transportation in packaging boxes. In addition, flavor compounds in peppers undergo changes during the drying process, resulting in unique characteristics that are distinct from those of fresh peppers, thereby enhancing the diversity of pepper products (Fernando & Amaratunga, 2022). Dried peppers are commonly used as spices in hot pots, soups, and other dishes. Moreover, they are often ground into a powder, typically used as a seasoning or ingredient in various food preparations (Liu et al., 2023).

A fresh pepper is covered with a thin layer of wax on its surface, which is the main barrier to moisture diffusion during drying. Traditional sun drying typically requires up to 21 days, is time-consuming, and difficult to control (Kaleemullah & Kailappan, 2005). To facilitate large-scale production of dried peppers and upgrade traditional sun-drying to controlled processes for producing quality-stable dried peppers, processing techniques have been developed and applied to commercial production. Thermal drying is a common and widely used method for drying pepper because of its simple operation, low equipment requirements, and low cost (Ozcan & Uslu, 2022; Yap, Uthairatanakij, Laohakunjit, & Jitareerat, 2022).

The primary goal of vegetable drying is to achieve an optimal texture, color, and flavor (Boateng, 2022). Temperature and duration, which are critical factors in the drying process, significantly affect the quality of the final product. Adjusting these parameters to achieve the desired dehydration level enables the production of high-quality products while reducing operational costs (Ikrang & Umani, 2019). From an engineering perspective, defining drying parameters is challenging because of the various physical, structural, chemical, and nutritional transformations that vegetables undergo during thermal drying. Therefore, an effective drying method must comprehensively consider the texture, color, and flavor of the vegetables. Therefore, this study aimed to investigate the effect of different drying durations on physico-chemical properties, microstructure, sensory traits, and volatile components of *Capsicum annuum* var. conoides. This study will also help to elucidate the drying mechanism of pepper and enhance its drying process.

## 2 Materials and methods

### 2.1 Chemicals

Capsaicin (≥98%) and dihydrocapsaicin (≥98%) were obtained from J&K Scientific Co., Ltd. (Beijing, China). Liquid chromatography-grade methanol, C7 − C30 n-alkane mixture, toluene‑*d*_8_ (≥99%, CAS 2037-26-5), and ethyl-d5 acetate-d3 (≥99%, CAS 117121–81-0) were obtained from Sigma-Aldrich Co., Ltd. (Shanghai, China). Sodium chloride (NaCl) was purchased from the China National Pharmaceutical Group Co. (Shanghai, China).

### 2.2 Sample preparation

Red pepper (*Capsicum annuum* var. conoides) cultivated and harvested in Guiyang, China, were refrigerated at 4 °C for up to 5 days before processing. The samples were visually inspected for color, size, and freshness to ensure the absence of mechanical damage. In the majority of Chinese factories, red peppers (*Capsicum annuum* var. conoides) are typically dried using hot air at 60 °C until their moisture content is below 10%. Subsequently, the samples were dried in a pilot-scale convection dryer at 60 °C until the moisture content remained <10% in this study. Fresh chili peppers as well as those dried for 200, 400, 600, 800, and 1000 min were selected for subsequent experiments.

### 2.3 Sensory assessment

A trained panel conducted sensory evaluations of the peppers. The panel comprised 12 individuals, 7 males and 5 females with an average age of 23.1 years, selected from Guizhou University. Prior to participation in the study, the panelists were proficient in quantitative descriptive analysis and identification of odor thresholds. Initially, the panelists referred to the literature to identify descriptors and reached a consensus on the most frequently used descriptors (Mamatha, Prakash, Nagarajan, & Bhat, 2008; Wang, MacRae, Wohlers, & Marsh, 2011; Zhu et al., 2023). Ultimately, six aroma descriptors were selected: fruity note, tingling aroma for choking note, fresh grass or vegetables for grassy note, woody aroma from cutting wood for dry wood note, cumin for herb note, and dry dust when wood is burned for smoky note. The panelists rated the intensity of each descriptor in each sample on a 5-point scale ranging from 0 (very weak) to 5 (very strong). Panelists underwent 10 sensory training sessions, which included odor standard solutions (“Le nez du vin,” Jean Lenoir, Provence, France) (Wang et al., 2023), fresh pepper, chili powder, chili oil, and dried chili samples. The evaluators' odor ratings were carefully monitored to ensure that they were approximately 20% of the group results (Zhu et al., 2023). Finally, the panelists evaluated both fresh and thermally dried pepper samples.

### 2.4 Surface color parameters

The color parameters (L*, a*, and b*) of fresh and pepper samples thermally dried for various durations were measured using a colorimeter (UltraScan Pro, Hunter Lab., Reston, VA, USA), according to the method outlined in Ref (Cai et al., 2023). L* characterizes the continuum between black and white on a scale of 0 (black) to 100 (white). a* delineates the gamut spanning from green to red, showcasing a range from −60 (indicating green) to 60 (indicating red). b* encapsulates the spectrum extending from blue to yellow, demonstrating values ranging from −60 (indicating blue) to 60 (indicating yellow) (Fante & Zapata Norena, 2012). The total color difference (*ΔE*) was calculated as a reference, representing the disparity in color between pepper samples subjected to no thermal drying and fresh ones (Wang et al., 2017).

### 2.5 Capsaicinoids quantitation

#### 2.5.1 Capsaicinoids extraction

Capsaicinoids were extracted from fresh and thermally dried peppers according to a previously described method with slight modifications (Vazquez-Espinosa et al., 2023). Pepper samples were pulverized using a micro ball mill (Type GT300, Beijing Grinder Instrument Co., Ltd., China). Then, 0.2 g of the pulverized sample was added to a centrifuge tube containing 15 mL methanol as the extraction solvent. The mixture was then exposed to a temperature of 50 °C in an ultrasonic bath operating at a frequency of 35 kHz for 5 h. The supernatant was centrifuged and concentrated under a gentle stream of nitrogen to obtain a final volume of 2.0 mL. The solution was then passed through a nylon membrane (13 mm, 0.22 μm) via filtration and preserved at 4 °C until further analysis.

#### 2.5.2 Capsaicinoids analysis by HPLC-DAD

The capsaicinoids analyse were conducted using HPLC-DAD (Agilent 1260, Agilent Technologies, USA) with a reversed-phase Agilent Zorbax SB-C18 column (4.6 × 250 mm, 5 μm). Elution was performed using an isocratic mixture of water and methanol (35:65, *v*/v). The flow rate was maintained at 1.0 mL/min, and the column temperature was regulated at 30 °C, with spectra recorded at a wavelength of 280 nm. The injection volume was 10 μL. Solutions containing capsaicin and dihydrocapsaicin were prepared by dissolving pure capsaicin or dihydrocapsaicin in methanol. Quantification was performed by comparing the retention times and peak areas using calibration curves established with external standards of capsaicin and dihydrocapsaicin (Gonzalez-Zamora et al., 2015).

### 2.6 Determination of Scoville heat units (SHU)

Once the capsaicinoid content is determined, it can be converted into Scoville heat units (SHU). The SHU was derived by multiplying the conversion coefficient of the capsaicinoids by their individual concentrations. The conversion coefficient for the capsaicin and dihydrocapsaicin content of the sample to SHU was 16.1 × 10^3^, whereas that for the remaining capsaicinoid content to SHU was 9.3 × 10^3^ (Zhang et al., 2023; Zhu et al., 2023).

### 2.7 Morphological properties and physical characteristics analysis

#### 2.7.1 Moisture content and thickness

The moisture content of the pepper samples was assessed by drying in an oven at 105 °C for 24 h, employing a gravimetric method (Demircan, Velioglu, & Giuffre, 2024; Yap et al., 2022). The thickness of the pepper samples was measured using a digital caliper (0.001 mm; Mitutoyo, Japan).

#### 2.7.2 Scanning electron microscope (SEM) observation

The microstructure of pepper samples was examined using a scanning electron microscope, following a reference protocol with modifications (Ahmadi, Zahedi, Ghorbanpour, & Mumivand, 2024). The best observation surface of the sample was chosen. Samples with a thickness of <2 mm were collected, and the surface was cleaned with ultrapure water to remove any contaminants, which ensured a pristine observation surface. The specimens were immersed in a solution consisting of 3% *v*/v glutaraldehyde and potassium phosphate buffer (0.05 M, pH 7.1) for 8 h to facilitate fixation. The fixed samples were gradually dehydrated with alcohol to replace water within the tissues. The samples were imaged using a scanning electron microscope (SEM; JSM-IT700HR, Japan).

### 2.8 Analysis of volatile compounds

The volatile composition of pepper samples at different stages of thermal drying, including the fresh state, was analyzed using headspace solid-phase microextraction coupled with gas chromatography mass spectrometry (HS-SPME-GC–MS). To ensure the representativeness of the volatile compounds, three peppers were selected as a group at each time point, mixed, pulverized using a micro ball mill (Type GT300, Beijing Grinder Instrument Co., Ltd., China), tested three times per group, and five groups were selected at each time point. Following previously described methods with slight modifications (Cuevas-Glory, Sosa-Moguel, Pino, & Sauri-Duch, 2015), 8 mg of pepper sample was placed in a 20-mL glass vial containing 2 mL of saturated NaCl. Additionally, the toluene‑*d*_8_ and ethyl-d5 acetate-d3 was added as an internal standard for semi-quantifying flavor compounds. An autosampler (Gerstel, Germany) equipped with a 50/30 μm DVB/CAR/PDMS fiber (2 cm; Supelco Inc., Bellefonte, PA) was used to extract volatile compounds from the headspace of the sample vial.

The sample was allowed to equilibrate at 40 °C for 5 min, followed by extraction for 30 min with stirring at 250 rpm. Subsequently, the fiber was transferred to the injection port and desorbed for 5 min at 250 °C using the unsplit mode. The GC–MS analysis was performed using a gas chromatograph (Agilent 7890, Agilent Technologies, USA) coupled to a mass spectrometer (Agilent 5977 B, Agilent Technologies, USA). Volatile compounds underwent separation and analysis on a DB-FFAP column (60 m × 0.25 mm i.d., 0.25 μm film thickness, J&W Scientific). The oven temperature program initiated at 40 °C for 1 min, ramped at 6 °C/min to 230 °C, and maintained at 230 °C for 15 min. Helium was used as the carrier gas at a constant flow rate of 2 mL/min. Mass spectra were acquired in electron ionization (EI) mode with an ionization energy of 70 eV. The ion source temperature was set at 230 °C, and the mass range scanned from 35 to 350 amu.

Volatile compound identification relies on simultaneously meeting the criteria of both mass spectrum and retention indices (RI). Compounds were identified based on the mass spectra, with a threshold set at 70% or higher similarity between the mass spectrometric data of each chromatographic peak and the National Institute of Standards and Technology (NIST) mass spectra library. The identification of compounds using RI involved comparison of their experimental RI with those reported in the scientific literature (RIL), ensuring that the difference remained ≤20 units (He et al., 2020).

### 2.9 Statistical analyses

Statistical analyses were performed using SPSS software (SPSS Inc., Chicago, IL, USA). Differences in significance among samples were assessed using one-way analysis of variance (ANOVA). Supervised partial least squares discriminant analysis (PLS-DA) was performed using SIMCA software (Umetrics, Umea, Sweden).

## 3 Results and discussion

### 3.1 Surface color

**Fig. 1:**
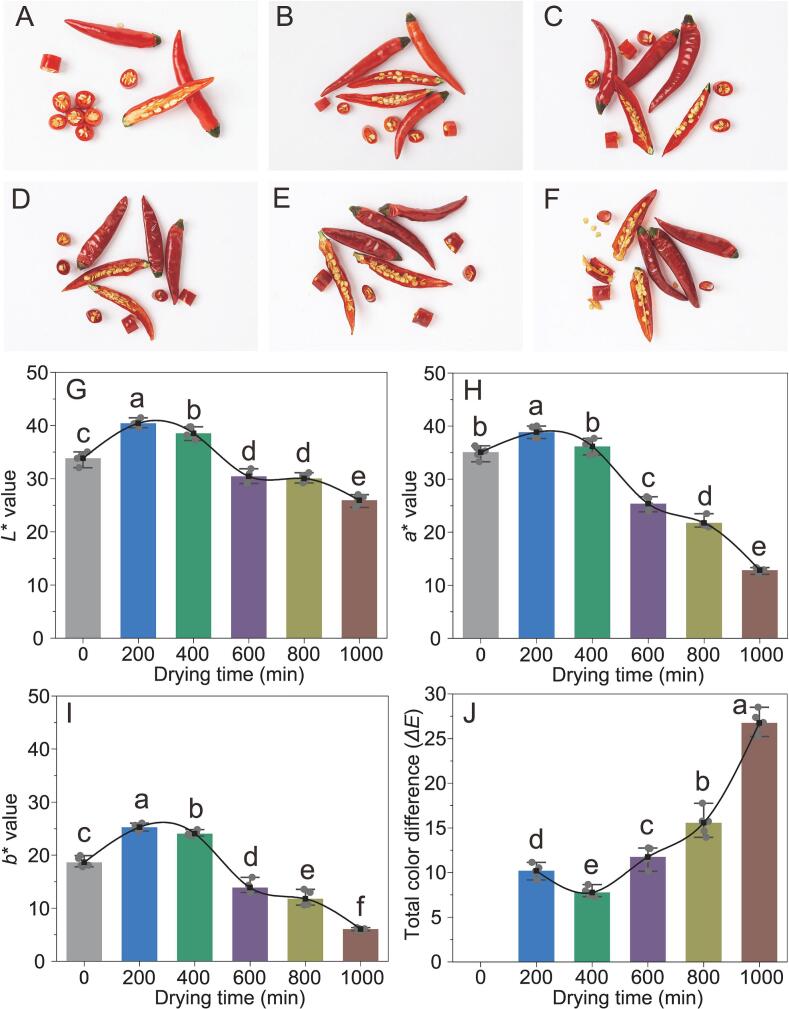
The visual changes during the pepper samples thermal drying process (A-F). Effect of different thermal drying times on the color characteristics (L*, a*, b* and *ΔE*) of pepper samples (G-J). Different letters represent significant differences (*p* < 0.05).

Color is a crucial organoleptic evaluation indicator for many agricultural products and significantly influences market value and sales volume (Pandiselvam et al., 2023). In this study, peppers thermally dried for different durations, as shown in Fig. 1A-F, can provide a visual sense of color changes. Color parameters (L*, a*, b*, *ΔE*) were used to quantitatively describe the color changes at different time intervals, as shown in Fig. 1G-J.

The initial L* value of pepper was 33.85, which decreased to 25.95 in the final stage of thermal drying, indicating a 23.3% reduction and gradual darkening of pepper color during thermal drying (Fig. 1G). The reduction in L* values may have resulted from the increased rate of non-enzymatic browning, including the Maillard reaction and caramelization, as well as the degradation of carotenoids during thermal drying (Vega-Galvez et al., 2009). A decrease in the a* value signifies a reduction in the intensity of the red color. Throughout the thermal-drying process, the a* value diminishes by 63.34% (Fig. 1H). Red carotenoids, primarily capsanthin and capsorubin, degrade at high temperatures (Daood, Kapitany, Biacs, & e., & Albrecht, K., 2006). Additionally, during thermal drying, processes such as the Maillard reaction led to pigment degradation, resulting in the dried peppers turning deep brown with decreasing a* values. The b* values exhibited a significant decrease of 67.5% during the thermal drying process, the most substantial decline among the three parameters L*, a*, and b*, suggesting a decrease in the yellow coloration of the pepper (Fig. 1I). Studies have suggested that a lower degree of yellowing of dried peppers enhances their overall quality (Ergüneş & Tarhan, 2006). The total color difference (*ΔE*) was assessed to compare the color between fresh and thermally dried chilies at various time intervals. According to Fig. 1J, a notable *ΔE* was observed between fresh and thermally dried samples (*p* < 0.05), with peppers undergoing thermal drying for 1000 min exhibiting the highest *ΔE*. Besides pigment degradation and the Maillard reaction, the increase in *ΔE* values may also be attributed to the impact of high thermal drying temperature and duration on heat-sensitive components such as proteins and carbohydrates (Wang et al., 2017). Thus, both non-enzymatic browning and the loss of heat-sensitive components likely contributed to the changes in the surface color of thermally dried peppers.

### 3.2 Moisture content, thickness and microstructure

**Fig. 2:**
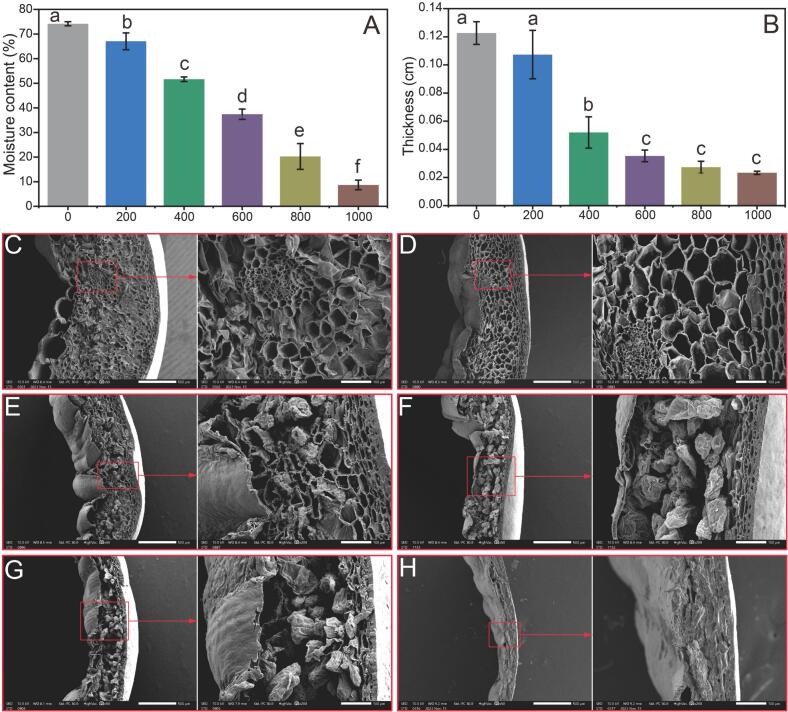
Effect of thermal drying times on moisture content (A), thickness (B), and microstructure (C—H) of pepper samples. Different letters represent significant differences (*p* < 0.05).

Moisture content is an important indicator of dried pepper. Dried peppers with excessive moisture content are susceptible to mold growth during storage. Conversely, excessively low moisture content leads to deterioration in flavor quality and increases susceptibility to breakage (Sasikumar, Mangang, Vivek, & Jaiswal, 2023). Currently, the moisture content of most dried pepper in China is <10%. Thus, the drying endpoint for pepper in this experiment was when the moisture content was below 10% (Fig. 2A). In the present study, the average initial moisture content of the pepper samples was 74.18%. Compared with the fresh green peppers reported in the literature, the fresh red peppers selected in this experiment had a relatively low moisture content (Zhang et al., 2023). As the thermal drying time increased, the moisture content gradually decreased, reaching only 8.69% after 1000 min. With an increase in the thermal drying time, the moisture continuously evaporated, and the thickness of the peppers decreased from an initial 0.123 cm to a final 0.023 cm at the end of thermal drying (Fig. 2B). Observing the changes in pepper thickness at the six selected time points, it was found that the thickness decreased most rapidly at 400 min, decreasing by 0.071 cm compared to the initial thickness. However, the changes in pepper thickness during the periods 0–200 min and 600–1000 min were not significant (*p* > 0.05). Scanning electron microscopy (SEM) was used to examine the influences of different thermal drying time on pepper surface properties (Fig. 2C-H). Variations in surface properties may indicate potential tissue damage, which may result in reduced nutritional value and alterations in pepper flavor (Lucic et al., 2023). During the initial drying stage (200 min), moisture evaporation increased the number of pores in the pepper samples (Fig. 2D). As the thermal drying duration increased, greater dehydration of the pepper resulted in internal tissue coalescing into small particles, whereas the inner surface exhibited cracking (Fig. 2F-G). A dense and firm texture developed during the final thermal drying stage (Fig. 2H).

### 3.3 Changes in capsaicinoids and pungent in peppers

**Fig. 3:**
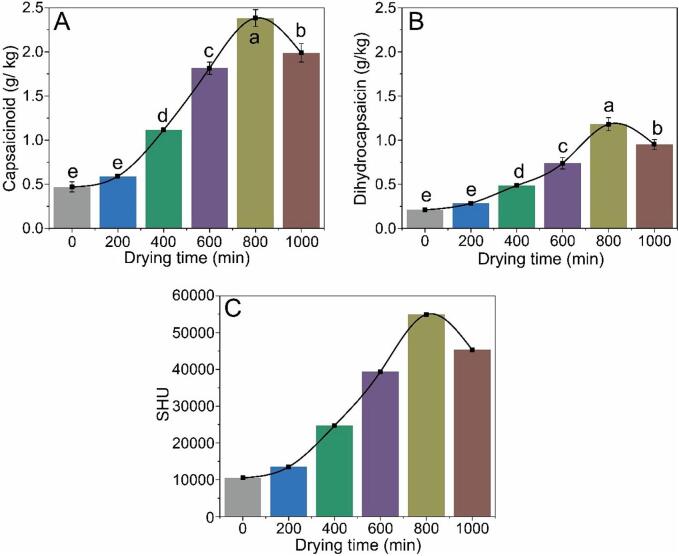
Effect of thermal drying times on capsaicin (A), dihydrocapsaicin (B), and SHU values (C) of pepper samples. Different letters represent significant differences (*p* < 0.05).

Capsaicinoids, including capsaicin and dihydrocapsaicin, are responsible for the pungency of peppers and constitute approximately 90% of the capsaicinoid compounds (Hamed, Kalita, Bartolo, & Jayanty, 2019). Fig. 3A-B presents the concentrations of capsaicin and dihydrocapsaicin in chili peppers. In fresh peppers, the concentrations of capsaicin and dihydrocapsaicin are 0.47 and 0.21 g/kg, respectively. After thermal drying for 200, 400, 600, 800, and 1000 min, the capsaicinoid contents were 0.59, 1.12, 1.82, 2.38, and 2.00 mg/kg, while the dihydrocapsaicin contents were 0.29, 0.49, 0.74, 1.18, and 0.95 mg/kg, respectively. Capsaicin content was significantly higher than that of dihydrocapsaicin (*p* < 0.05). Thermal drying significantly increased the capsaicinoid content in peppers. Thermal drying leads to rapid dehydration of peppers, which primarily contributes to an increase in capsaicin content. However, when the heating time reached 800 min, the capsaicin content began to decrease, mainly because capsaicin underwent alkyl cleavage upon exposure to high temperatures for extended periods, forming vanillin, 8-methyl-6-nonenoamide, and 8-methyl-6-nonenoic acid via further oxidation (Henderson & Henderson, 1992; Mba, Dumont, & Ngadi, 2017).

The pungency of the peppers was measured using the SHU, which correlates with capsaicinoid content. This index categorizes pungency into five levels: non-pungent (0–700 SHU), low (700–3000 SHU), moderate (3000–25,000 SHU), high (25000–75,000 SHU), and very high (>80,000 SHU) (Al Othman, Ahmed, Habila, & Ghafar, 2011; Karaman, Pinar, Ciftci, & Kaplan, 2023). The SHU values are shown in Fig. 3C. The results showed that the pungency of the peppers varied with thermal drying time. The spiciness levels of dried peppers were higher than those of fresh peppers. An SHU value of 10,486 for fresh peppers indicated moderate pungency. When the drying time was increased to 600 min, the SHU values all exceed 25,000, reaching a high level of spiciness.

### 3.4 Sensory properties

**Fig. 4:**
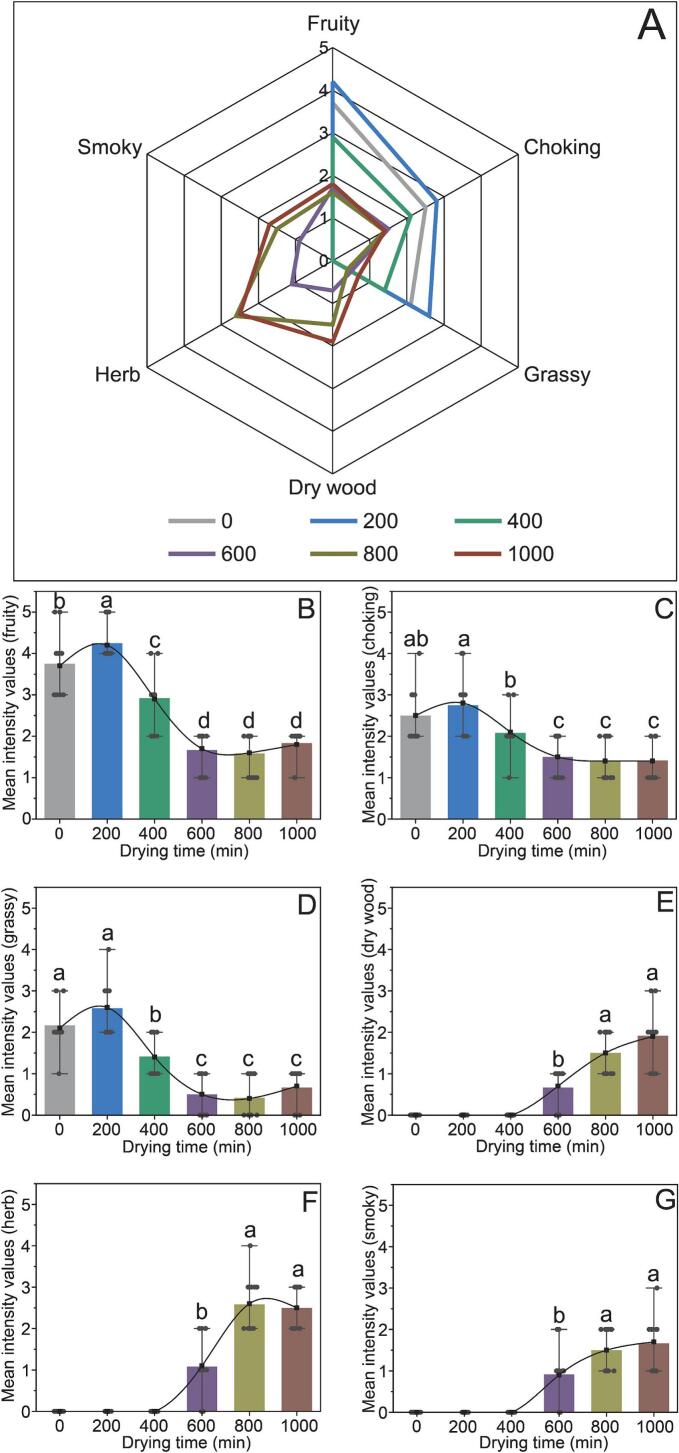
Aroma profiles of pepper samples under different thermal drying times (A). Effect of thermal drying times on fruity (B), choking (C), grassy (D), dry wood (E), herb (F), and smoky (G) notes of pepper samples. Different letters represent significant differences (*p* < 0.05).

Sensory evaluation of the pepper was conducted for various thermal drying durations. Six descriptors, namely fruity, choking, grassy, dry wood, herb, and smoky, were employed to characterize the aroma volatilization process during thermal drying. Fig. 4A illustrates the average sensory scores of the peppers after 0 and 1000 min of thermal drying. In fresh peppers, only the sensory attributes fruity, choking, and grassy are evident, with the highest sensory intensity observed at 200 min of thermal drying (Fig. 4B-D), which may be attributed to the initial drying phase where high temperatures prompt moisture evaporation in peppers, leading to an elevation in aroma compound concentration and subsequently sensory intensity. However, the changes in these three sensory attributes during the late thermal drying stage (600–1000 min) were not significant (*p* > 0.05). The sensory attributes of dry wood, herb, and smoky emerge after 400 min of drying (Fig. 4E-G), exhibiting a progressive increase in sensory intensity with prolonged drying, likely influenced by continual high temperatures stimulating chemical reactions and generating a more complex aroma profile. In summary, compared to fresh peppers, drying confers additional sensory attributes to peppers, with the attributes of dry wood, herb, and smoky prevailing in the final dried peppers. Statistical analysis revealed significant differences in the intensity values of pepper across various time points for all descriptors (*p* < 0.05).

### 3.5 The types of volatile compounds during thermal drying in peppers

**Fig. 5:**
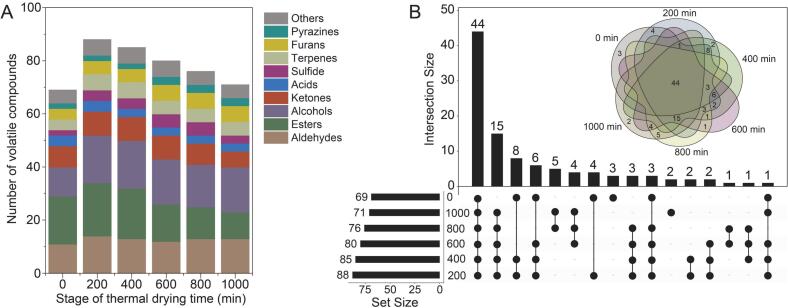
The number of volatile compounds in pepper samples under different thermal drying times (A) and the Venn diagram illustrating the similarities and differences in these compounds (B).

A total of 104 volatile compounds were identified in the pepper samples during thermal drying, including 22 esters, 20 alcohols, 18 aldehydes, 12 ketones, 7 terpenes, 6 furans, 5 acids, 5 sulfides, 3 pyrazines, and 6 other volatile compounds. The volatile compounds present in the pepper samples at six different thermal drying stages are listed in Supplementary Table S1. Fig. 5A shows that the types of volatile compounds increased in the early stages of thermal drying, which may be due to high temperatures causing compounds to undergo processes such as oxidation, degradation, and the Maillard reaction to generate additional compounds. However, with continued high-temperature heating, the types of compound decomposition decreased. Esters, alcohols, and aldehydes are the most abundant compounds at each stage, together comprising over 50% of all volatile compounds. Among the esters, ethyl esters, including ethyl acetate, ethyl propionate, ethyl butanoate, ethyl hexanoate, were predominant. These ethyl esters are key aroma compounds in some common fruits such as pineapple, apple, banana, and kiwifruit, imparting the characteristic fragrance to peppers. A total of 44 volatile compounds were detected at each heating stage (Fig. 5B), accounting for 42.3% of all compounds, and these 44 common volatile compounds may include aroma compounds that constitute the six fixed sensory attributes of peppers during the drying stage (Fig. 4A). The high overlap in the Venn diagram indicated that most volatile compounds were shared across multiple drying stages.

### 3.6 Variation pattern of volatile compounds during thermal drying

**Fig. 6:**
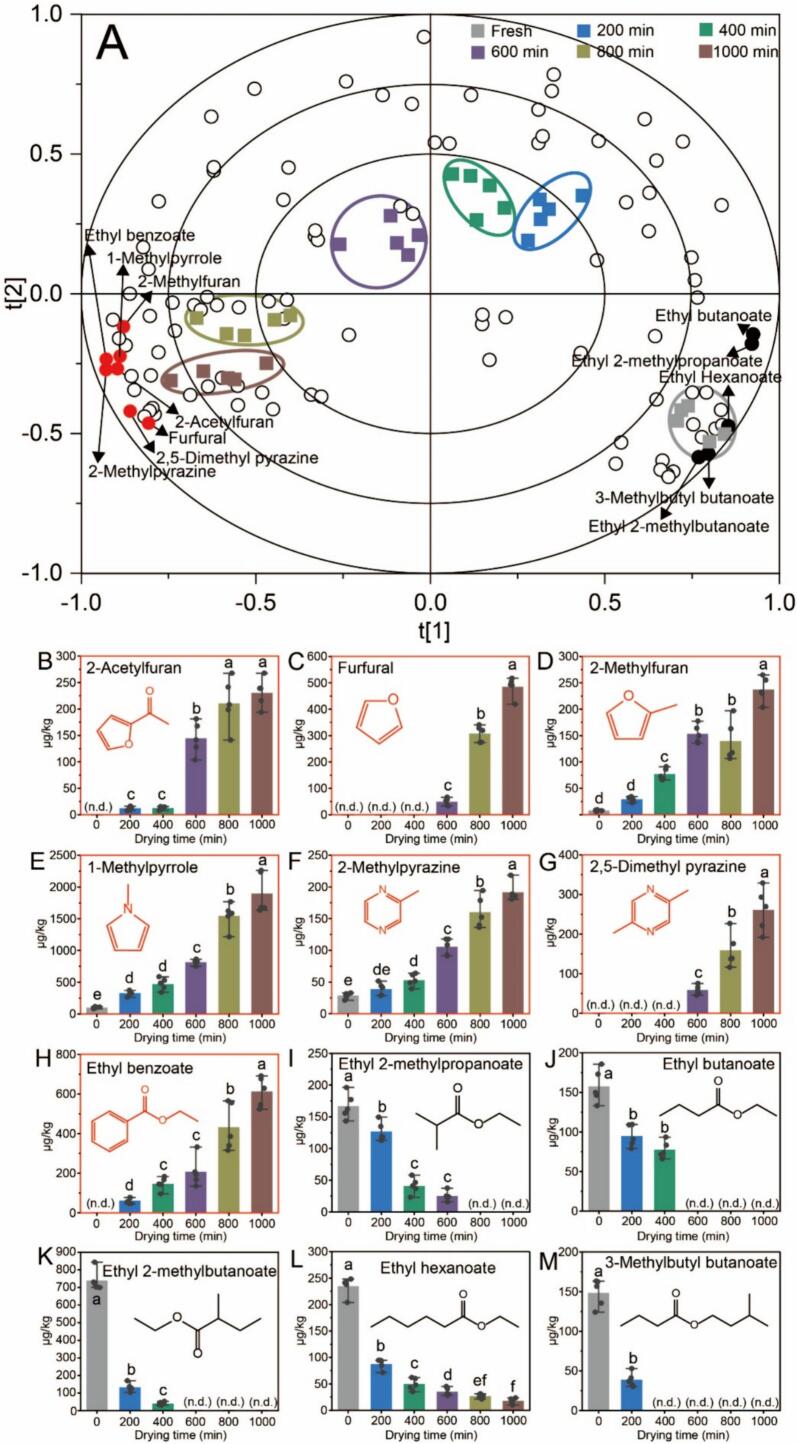
The PLS-DA model graph depicts the volatile compound profiles (A), and the relative concentrations of volatile compound in pepper samples under different thermal drying times (B-M). Different letters represent significant differences (*p* < 0.05).

Significant changes occurred in the types and concentrations of volatile compounds during the thermal drying of peppers. Studying the objective alterations in these compounds serves as the foundation for understanding the changes in the sensory properties of peppers. Multivariate statistical analysis was used to develop mathematical models to establish the relationships between the volatile compounds and thermal drying times. Partial least-squares discriminant analysis (PLS-DA) was applied to determine the associations between thermal drying time (y variables, *n* = 6) and volatile compounds (x variables, *n* = 104) in pepper samples subjected to thermal drying (Fig. 6A). The PLS-DA model established using volatile compounds exhibited R2Y and Q2 values of 0.958 and 0.837, respectively, indicating good stability and predictive ability (Xie et al., 2020; Yang, Fan, & Xu, 2021). This model enables the identification and differentiation of pepper samples with varying thermal drying times. These results indicate that peppers at different thermal drying time points contain characteristic volatile compounds and that identifying markers during the production process is important for quality monitoring. Based on analysis of the variable importance for projection (VIP) values from the PLS-DA model and *P* values from the quantification results (He et al., 2020), some heterocyclic compounds and esters exhibited certain patterns during the thermal drying process. The concentrations of 2-acetylfuran, furfural, 2-methylfuran, 1-methylpyrrole, 2-methylpyrazine, 2,5-dimethylpyrazine, and ethyl benzoate were positively correlated with the thermal drying time (Fig. 6B-H), while those of ethyl 2-methylpropanoate, ethyl butanoate, ethyl 2-methylbutanoate, ethyl hexanoate, and 3-methylbutyl butanoate were negatively correlated with the thermal drying time (Fig. 6I-M).

Furan is found in various thermally processed foods such as baked, fried, and roasted foods (Juaniz, Zocco, Mouro, Cid, & Paz De Pena, 2016). The formation of furan in these foods may result from different pathways, including thermal degradation or the rearrangement of sugars, ascorbic acid, and amino acids, as well as the oxidation of polyunsaturated fatty acids (Batool, Chen, Liu, Chen, & Wang, 2023). Fig. 6B-D illustrates a significant increase in the concentrations of 2-acetylfuran, furfural, and 2-methylfuran during thermal drying. Fresh pepper (0 min) did not contain 2-acetylfuran and furfural. However, after 1000 min of thermal drying, the content of 2-acetylfuran reached 210.44 μg/kg and furfural reached 484.78 μg/kg. After heating for 1000 min, the content of 2-methylfuran increased from 7.93 μg/kg to 237.13 μg/kg. The concentrations of 1-methylpyrrole, 2-methylpyrazine, and 2,5-dimethylpyrazine in the heterocyclic compounds increased linearly with thermal drying time (Fig. 6E-G). After 1000 min of thermal drying, the concentrations of 1-methylpyrrole and 2-methylpyrazine increased by 19 and 7 times respectively, compared to those in fresh peppers, whereas 2,5-dimethylpyrazine was only detected after 600 min of thermal drying. Pyrazine and pyrrole are commonly found in foods processed at high temperatures and are common products of the Maillard reaction. After drying, volatile heterocyclic compounds with baking aroma, such as pyrrole and pyrazine, can significantly enhance the sensory properties of tea (Wang et al., 2024), bread (Pico, Khomenko, Capozzi, Navarini, & Biasioli, 2020), and coffee (Cao, Wu, Viejo, Dunshea, & Suleria, 2023) via the Maillard reaction.

The concentrations of ethyl 2-methylpropanoate, ethyl butanoate, ethyl 2-methylbutanoate, ethyl hexanoate, and 3-methylbutyl butanoate gradually decreased with increasing thermal drying time (Fig. 6I-M). These ester compounds impart typical fruity flavors reminiscent of apples, pineapples, and kiwifruits; however, they may substantially decline in quantity during the later stages of thermal drying owing to poor heat stability. Ethyl 2-methylpropanoate, ethyl butanoate, ethyl 2-methylbutanoate, and 3-methylbutyl butanoate were undetectable in pepper during the later stages of thermal drying. Similar results were also observed in the sensory studies (Fig. 4B), with typical fruity aromas in fresh peppers, but significantly reduced fruity aromas in dried peppers, which is possibly correlated with the decreased concentration of ester compounds. Notably, ethyl benzoate (Fig. 6H) exhibited an inverse relationship with the other esters. Undetected in fresh peppers, its concentration increased with thermal drying time, potentially forming benzene ring compounds at high temperatures, thereby associating with degraded ester compounds.

## 4 Conclusions

This study examined the physicochemical and sensory changes in peppers (*Capsicum annuum* var. conoides) during the thermal drying process. Throughout the drying process, extended exposure to high temperatures led to considerable darkening, with a noticeable reduction in the red and yellow hues. The pepper microstructure shifted from granular to compact after dehydration, with a decrease in thickness of 81% and a moisture content of 88%. Capsaicin and dihydrocapsaicin, which are the major contributors to pepper spiciness, increased during thermal drying to 3.2- and 3.5-times the fresh pepper levels, respectively. The sensory profile shifted from grassy, fruity, and choked to herbal, dry wood, and smoky. The grassy, fruity, and choking attributes decreased by 69%, 51%, and 43%, respectively, whereas the herb, dry wood, and smoky attributes were imperceptible in fresh peppers but reached sensory intensities of 2.5, 1.9, and 1.7 after thermal drying. A total of 104 volatile compounds were identified, of which 44 were detected after thermal drying. The ethyl 2-methylpropanoate, ethyl butanoate, ethyl 2-methylbutanoate, ethyl hexanoate, and 3-methylbutyl butanoate contents gradually decreased with increasing thermal drying time, whereas those of 2-acetylfuran, furfural, 1-methylpyrrole, 2-methylpyrazine, and 2,5-dimethylpyrazine increased. These findings offer insights for monitoring physicochemical parameters, optimizing processes, and adjusting flavors during thermal drying of pepper.

## Ethical statement

Ethical review and approval were waived for this study, because experimental samples used in the study are consumed in daily life.

## Informed consent statement

Informed consent was obtained from all panelists involved in the study. The participants have given permission to participate and use their data/answers. It is affirmed that the research was executed in strict adherence to protocols designed to safeguard the rights and privacy of all participants.

## CRediT authorship contribution statement

**Cen Li:** Writing – review & editing, Writing – original draft, Validation, Methodology, Investigation. **Yongjun Wu:** Writing – review & editing, Resources, Project administration, Funding acquisition. **Qiyan Zhu:** Methodology, Investigation. **Chuanzheng Xie:** Resources, Project administration, Investigation. **Yan Yan:** Writing – review & editing, Writing – original draft, Investigation.

## Declaration of competing interest

The authors declare that they have no known competing financial interests or personal relationships that could have appeared to influence the work reported in this paper.

## Data Availability

Data will be made available on request.

## Appendix A Supplementary data

Supplementary material Image 1
