# Supplementary material for: Alterations in physico-chemical properties, microstructure, sensory characteristics, and volatile compounds of red pepper (Capsicum annuum var. conoides) during various thermal drying durations
Source: Food Chem X. 2024 Jun 20;23:101566. doi: 10.1016/j.fochx.2024.101566 (PMC11245976; doi:10.1016/j.fochx.2024.101566)
Supplement: Supplementary file 1 — Supplementary material [file mmc1.docx]

Supplementary Material

Table S1. Volatile compounds of peppers at six thermal drying stages.

| **No.** | **Compounds** | **CAS** | **RI ^a^** | **RIL ^b^** | **Stage of thermal drying time** | | | | | |
| --- | --- | --- | --- | --- | --- | --- | --- | --- | --- | --- |
|  |  |  |  |  | **0** | **200** | **400** | **600** | **800** | **1000** |
| *Aldehydes* | | | | | | | | | | |
| 1 | Acetaldehyde | 75-07-0 | 692 | 686 | × | √ | √ | √ | √ | √ |
| 2 | 2-Methylpropanal | 78-84-2 | 819 | 813 | × | × | × | × | √ | √ |
| 3 | 2-Propenal | 107-02-8 | 830 | 838 | √ | √ | √ | √ | √ | √ |
| 4 | Butanal | 123-72-8 | 875 | 867 | √ | √ | √ | √ | √ | √ |
| 5 | 2-Methylbutyraldehyde | 96-17-3 | 890 | 880 | √ | √ | √ | √ | √ | √ |
| 6 | Isovaleraldehyde | 590-86-3 | 919 | 912 | √ | √ | √ | √ | √ | √ |
| 7 | Hexanal | 66-25-1 | 1071 | 1083 | √ | √ | √ | √ | √ | √ |
| 8 | 2-Pentenal | 764-39-6 | 1090 | 1087 | √ | √ | √ | √ | × | × |
| 9 | 3-Hexenal | 4440-65-7 | 1132 | 1146 | √ | √ | × | × | × | × |
| 10 | Heptanal | 111-71-7 | 1189 | 1181 | √ | √ | √ | √ | √ | √ |
| 11 | 3-Methyl-2-butenal | 107-86-8 | 1210 | 1222 | × | × | × | × | √ | √ |
| 12 | 2-Hexenal | 505-57-7 | 1243 | 1248 | √ | √ | √ | √ | √ | √ |
| 13 | Octanal | 124-13-0 | 1290 | 1291 | × | √ | √ | × | × | × |
| 14 | Nonanal | 124-19-6 | 1381 | 1390 | × | √ | √ | √ | × | × |
| 15 | Benzaldehyde | 100-52-7 | 1538 | 1529 | √ | √ | √ | √ | √ | √ |
| 16 | 2-Nonenal | 2463-53-8 | 1527 | 1536 | × | × | × | × | √ | √ |
| 17 | 2,6-Nonadienal | 17587-33-6 | 1727 | 1715 | √ | √ | √ | √ | × | × |
| 18 | Benzeneacetaldehyde | 122-78-1 | 1655 | 1650 | × | × | × | × | √ | √ |
| *Esters* | | | | | | | | | | |
| 19 | Methyl acetate | 79-20-9 | 827 | 827 | × | √ | √ | √ | √ | √ |
| 20 | Ethyl Acetate | 141-78-6 | 885 | 883 | √ | √ | √ | × | × | × |
| 21 | Ethyl propionate | 105-37-3 | 953 | 949 | √ | √ | √ | √ | √ | √ |
| 22 | Ethyl 2-methylpropanoate | 97-62-1 | 969 | 965 | √ | √ | √ | √ | × | × |
| 23 | Propyl acetate | 109-60-4 | 980 | 973 | √ | √ | √ | √ | √ | √ |
| 24 | Ethyl butanoate | 105-54-4 | 1052 | 1048 | √ | √ | √ | × | × | × |
| 25 | Ethyl 2-methylbutanoate | 7452-79-1 | 1058 | 1062 | √ | √ | √ | × | × | × |
| 26 | Ethyl 3-methylbutanoate | 108-64-5 | 1079 | 1077 | √ | √ | × | × | × | × |
| 27 | 3-Methylbutyl acetate | 123-92-2 | 1223 | 1226 | √ | √ | √ | × | × | × |
| 28 | Ethyl 4-methylpentanoate | 25415-67-2 | 1199 | 1193 | √ | √ | √ | √ | √ | √ |
| 29 | Ethyl hexanoate | 123-66-0 | 1249 | 1241 | √ | √ | √ | √ | √ | √ |
| 30 | 3-Methylbutyl butanoate | 106-27-4 | 1266 | 1256 | √ | √ | × | × | × | × |
| 31 | 3-Methylbutyl 2-methylbutanoate | 27625-35-0 | 1279 | 1276 | √ | √ | √ | √ | √ | √ |
| 32 | 3-Methylbutyl 3-methylbutyrate | 659-70-1 | 1290 | 1283 | √ | √ | √ | × | × | × |
| 33 | Pentyl 2-methylbutanoate | 68039-26-9 | 1316 | 1316 | × | × | √ | √ | √ | × |
| 34 | Hexyl 2-methylpropanoate | 2349-07-7 | 1347 | 1335 | √ | √ | √ | × | × | × |
| 35 | Pentyl 3-methylbutyrate | 25415-62-7 | 1361 | 1350 | √ | √ | √ | √ | × | × |
| 36 | Hexyl 2-methylbutanoate | 10032-15-2 | 1411 | 1418 | √ | √ | √ | √ | √ | √ |
| 37 | Ethyl octoate | 106-32-1 | 1437 | 1431 | × | × | × | √ | √ | √ |
| 38 | Hexyl 3-methylbutanoate | 10032-13-0 | 1449 | 1447 | √ | √ | √ | √ | √ | × |
| 39 | Ethyl benzenecarboxylate | 93-89-0 | 1668 | 1652 | × | √ | √ | √ | √ | √ |
| 40 | Methyl salicylate | 119-36-8 | 1770 | 1756 | √ | √ | √ | √ | √ | √ |
| *Alcohols* | | | | | | | | | | |
| 41 | 2-Butanol | 78-92-2 | 1025 | 1027 | √ | √ | √ | √ | √ | √ |
| 42 | 1-Propanol | 71-23-8 | 1043 | 1038 | × | √ | √ | √ | √ | √ |
| 43 | 2-Methyl-1-propanol | 78-83-1 | 1111 | 1101 | × | √ | √ | √ | √ | √ |
| 44 | 3-Pentanol | 584-02-1 | 1119 | 1110 | × | √ | √ | × | × | × |
| 45 | 1-Methoxypropan-2-ol | 107-98-2 | 1171 | 1160 | × | √ | √ | √ | √ | √ |
| 46 | 1-Butanol | 71-36-3 | 1175 | 1160 | × | √ | √ | √ | √ | × |
| 47 | 1-Penten-3-ol | 616-25-1 | 1177 | 1165 | √ | √ | √ | √ | √ | √ |
| 48 | 3-Methyl-1-butanol | 123-51-3 | 1221 | 1211 | √ | √ | √ | √ | √ | √ |
| 49 | 2-Ethoxyethanol | 110-80-5 | 1248 | 1239 | × | × | × | × | × | √ |
| 50 | 1-Pentanol | 71-41-0 | 1276 | 1261 | √ | √ | √ | √ | √ | √ |
| 51 | 4-Methyl-1-pentanol | 626-89-1 | 1315 | 1311 | √ | √ | √ | √ | √ | √ |
| 52 | 1-Hexanol | 111-27-3 | 1352 | 1359 | √ | √ | √ | √ | × | √ |
| 53 | 4-Methyl-3-penten-1-ol | 763-89-3 | 1380 | 1390 | √ | √ | √ | √ | √ | √ |
| 54 | Cyclohexanol | 108-93-0 | 1411 | 1403 | × | × | × | √ | √ | √ |
| 55 | 4-Methyl-1-hexanol | 818-49-5 | 1439 | 1434 | √ | √ | √ | √ | √ | √ |
| 56 | 1-Octen-3-ol | 3391-86-4 | 1467 | 1456 | √ | √ | √ | × | × | × |
| 57 | 1-Octanol | 111-87-5 | 1577 | 1565 | × | √ | √ | √ | √ | √ |
| 58 | 2-Decanol | 1120-06-5 | 1589 | 1585 | × | √ | √ | √ | √ | √ |
| 59 | Benzyl alcohol | 100-51-6 | 1898 | 1885 | √ | √ | √ | √ | √ | √ |
| 60 | Phenylethyl Alcohol | 60-12-8 | 1912 | 1912 | √ | √ | √ | √ | √ | √ |
| *Ketones* | | | | | | | | | | |
| 61 | 3-Methyl-2-butanone | 563-80-4 | 928 | 929 | √ | √ | √ | √ | × | × |
| 62 | 2-Butanone | 78-93-3 | 945 | 940 | √ | √ | √ | √ | √ | √ |
| 63 | 3-Pentanone | 96-22-0 | 979 | 974 | √ | √ | √ | √ | √ | × |
| 64 | 2-Methyl-3-pentanone | 565-69-5 | 1011 | 1000 | × | √ | √ | √ | √ | × |
| 65 | 1-Penten-3-one | 1629-58-9 | 1025 | 1019 | √ | √ | √ | √ | √ | × |
| 66 | 2,3-Pentanedione | 600-14-6 | 1073 | 1062 | √ | √ | √ | √ | × | × |
| 67 | 3-Penten-2-one | 625-33-2 | 1134 | 1121 | × | × | × | × | √ | √ |
| 68 | Acetoin | 513-86-0 | 1297 | 1287 | × | √ | √ | √ | √ | √ |
| 69 | 6-Methyl-5-hepten-2-one | 110-93-0 | 1350 | 1342 | √ | √ | √ | √ | √ | √ |
| 70 | 2,3-dimethyl-2-cyclopentenone | 1121-05-7 | 1533 | 1524 | √ | √ | √ | √ | √ | √ |
| 71 | 2(3H)-Furanone | 96-48-0 | 1626 | 1617 | × | × | × | × | × | √ |
| 72 | Geranylacetone | 3796-70-1 | 1855 | 1862 | √ | × | × | × | × | × |
| *Acids* | | | | | | | | | | |
| 73 | Acetic acid | 64-19-7 | 1446 | 1448 | √ | √ | √ | √ | √ | √ |
| 74 | 2-Methylbutanoic acid | 116-53-0 | 1663 | 1668 | × | √ | √ | √ | √ | √ |
| 75 | 3-Methylbutanoic acid | 503-74-2 | 1676 | 1678 | √ | √ | × | × | × | × |
| 76 | 4-Methylpentanoic acid | 646-07-1 | 1831 | 1820 | √ | √ | √ | √ | √ | √ |
| 77 | 2-Methylpropanoic acid | 79-31-2 | 1570 | 1576 | √ | × | × | × | × | × |
| *Sulfide* | | | | | | | | | | |
| 78 | Dimethyl sulfide | 75-18-3 | 572 | 757 | √ | √ | √ | √ | √ | √ |
| 79 | Dimethyl disulfide | 624-92-0 | 1064 | 1066 | × | √ | √ | √ | √ | √ |
| 80 | Methyl 2-propenyl disulfide | 2179-58-0 | 1260 | 1266 | × | √ | √ | √ | √ | × |
| 81 | Methional | 3268-49-3 | 1460 | 1469 | √ | √ | √ | √ | √ | √ |
| 82 | Diallyl disulphide | 2179-57-9 | 1468 | 1470 | × | × | × | √ | √ | × |
| *Terpenes* | | | | | | | | | | |
| 83 | Limonene | 138-86-3 | 1209 | 1198 | √ | √ | √ | √ | √ | √ |
| 84 | Eucalyptol | 470-82-6 | 1217 | 1212 | √ | √ | √ | √ | √ | √ |
| 85 | Linalool | 78-70-6 | 1544 | 1552 | √ | √ | √ | × | × | × |
| 86 | β-Elemene | 515-13-9 | 1587 | 1598 | × | √ | √ | √ | √ | √ |
| 87 | β-Cyclocitral | 432-25-7 | 1597 | 1599 | √ | × | × | × | × | × |
| 88 | β-Cedrene | 546-28-1 | 1620 | 1611 | × | √ | √ | √ | √ | √ |
| 89 | β-Lonone | 79-77-6 | 1934 | 1943 | × | √ | √ | √ | √ | √ |
| Furans | | | | | | | | | | |
| 90 | Furan | 110-00-9 | 795 | 797 | √ | √ | √ | √ | √ | √ |
| 91 | 2-Methylfuran | 534-22-5 | 877 | 876 | √ | √ | √ | √ | √ | √ |
| 92 | 2-Ethylfuran | 3208-16-0 | 969 | 960 | √ | √ | √ | √ | √ | √ |
| 93 | 2-Pentylfuran | 3777-69-3 | 1241 | 1230 | √ | √ | √ | √ | √ | √ |
| 94 | Furfural | 98-01-1 | 1448 | 1459 | × | × | × | √ | √ | √ |
| 95 | 2-Acetylfuran | 1192-62-7 | 1511 | 1501 | × | √ | √ | √ | √ | √ |
| Pyrazines | | | | | | | | | | |
| 96 | Pyrazine | 290-37-9 | 1222 | 1223 | √ | √ | √ | √ | √ | √ |
| 97 | 2-Methylpyrazine | 109-08-0 | 1284 | 1271 | √ | √ | √ | √ | √ | √ |
| 98 | 2,5-Dimethyl pyrazine | 123-32-0 | 1334 | 1323 | × | × | × | √ | √ | √ |
| Others | | | | | | | | | | |
| 99 | 1,1-Diethoxyethane | 105-57-7 | 892 | 899 | √ | √ | √ | √ | √ | √ |
| 100 | 1-Methylpyrrole | 96-54-8 | 1135 | 1140 | √ | √ | √ | √ | √ | √ |
| 101 | Thiazole | 288-47-1 | 1258 | 1249 | × | √ | √ | √ | × | × |
| 102 | Styrene | 100-42-5 | 1266 | 1250 | √ | √ | √ | √ | √ | √ |
| 103 | 2-methoxyphenol | 90-05-1 | 1877 | 1860 | √ | √ | √ | √ | √ | √ |
| 104 | Phenol | 108-95-2 | 2009 | 2004 | √ | √ | √ | √ | √ | √ |

^a^ linear retention index on a DB-WAX column.

^b^ Retention index of the literature on DB-Wax capillary column (https://webbook.nist.gov/chemistry/cas-ser/).
